# Reduced Growth and Inflammation in *Lrp5* ^*−/−*^ Mice Adipose Tissue

**DOI:** 10.1111/jcmm.70670

**Published:** 2025-10-13

**Authors:** Aureli Luquero, Noelia Pimentel, Gemma Vilahur, Lina Badimon, Maria Borrell‐Pages

**Affiliations:** ^1^ Molecular Pathology and Therapeutic of Ischemic and Atherothrombotic Diseases Sant Pau Research Institute (IR‐Sant Pau) Barcelona Spain; ^2^ Biomedicine Doctorate Program, Universitat de Barcelona Barcelona Spain; ^3^ CIBER‐CV, Instituto de Salud Carlos III Madrid Spain

**Keywords:** adipose tissue, canonical WNT signalling, immune infiltration, inflammation, lipoprotein receptor

## Abstract

Obesity is a major cause of chronic disease morbidity–mortality. Understanding the mechanisms triggering its promotion will allow the development of effective treatments. Cardiovascular diseases are associated with high‐fat diet ingestion; however, how adiposity is distributed in relation to fat intake is not known. The lipoprotein receptor LRP5 participates in lipid handling in several cells. Whether LRP5 and its effector canonical WNT signalling pathway are involved in high‐fat diet‐induced adipose tissue distribution remains unknown. We fed *Wt* and *Lrp5* ^*−/−*^ mice with a high cholesterol diet and analysed adipose and inflammatory markers. More fat deposition in *Wt* mice than in *Lrp5* ^*−/−*^ mice is observed upon high‐fat diet intake. Lipoprotein receptor expression is increased in mice visceral and subcutaneous adipose tissues of hypercholesterolemic mice. Gene expression markers of adiposity and inflammation show that LRP5 deficiency reduces adipocyte growth and differentiation while decreasing macrophage infiltration. LRP5 and LRP1 gene expression are also increased in human adipose tissues of obese patients, further suggesting that lipoprotein receptors participate in adipose tissue growth. In conclusion, LRP5 induces adipocyte proliferation and insulin sensitivity and, simultaneously, enhances macrophage's infiltrating capacity, triggering the inflammatory process associated with proliferating adipose tissues. This study shows that therapies can arise from research on canonical WNT signalling in adipose tissues to prevent obesity.

## 1 Introduction

The relationship between obesity and cardiovascular diseases is undeniable. Weight gain in obese humans is characterised by adipose tissue hypertrophy that eventually leads to cardiovascular diseases. Before the year 2000, to evaluate obesity, researchers focused mainly on the amount of adipose tissue. However, recent studies demonstrate that adipose tissue distribution has a major role in the determination of cardiovascular risk [1, 2, 3, 4]. Adipose tissues have unique properties and can be classified into white adipose tissue (WAT) and brown adipose tissue (BAT). While WAT's main function is to serve as a fat deposit, BAT is metabolically more active as its main function is to regulate body temperature [5, 6]. As BAT metabolism favours energy expenditure, high BAT adiposity is associated with better cardiovascular health [7].

The prevalence of childhood obesity has increased worldwide over the last decades. In individuals aged 5–19 years, the percentage of obese females rose from 0.7% in 1975 to 5.6% in 2016, while in males the percentage increased from 0.9% to 7.8% over the same period [8]. Although since the year 2000 the mean body‐mass index stopped increasing in high‐income countries, the growth in low‐ and middle‐income countries has persisted, which, together with the increasing human population, turns obesity into a public health problem worldwide [9].

The two major reservoirs of WAT in adulthood are subcutaneous and abdominal fat. Subcutaneous white adipose tissue (SCAT) is the physiologic buffer for excess energy intake (hypercaloric diets), absorbing circulating sugars and fats in response to insulin and generating triglycerides that are stored in adipocytes [10]. When the storage capacity of SCAT is exceeded, fat starts to accumulate as visceral adipose tissue (VAT) in different regions of the abdominal cavity [11]. In normal conditions, SCAT is approximately 80%–90% of the total WAT in human adults, while VAT accounts for 10%–20% of total WAT in men and 5%–8% in women [12, 13]. The amount of VAT increases with age in both genders [12].

Increased VAT but not SCAT induces increased risk of diabetes, elevated circulating cholesterol and triglycerides, hypertension, metabolic syndrome, stroke, peripheral artery disease, and/or reduced thickness in vascular walls [14, 15, 16]. This is because there is higher innervation, irrigation, and vascularisation in VAT compared to SCAT, connecting the fat depot to systemic organ function by nerves and hormones [14]. As VAT becomes more vascularised, inflammatory cells infiltrate VAT more easily, conferring higher inflammatory properties to the tissue [17]. VAT is more sensitive than SCAT in response to glucocorticoid‐, catecholamine‐ and androgen‐stimulus, but SCAT reacts preferentially to oestrogen stimulus [18].

Epicardial adipose tissue (EAT) is the WAT tissue that covers the heart, being more abundant in the atrioventricular node, the interventricular grooves, and the right ventricle, where it protects the myocardium [19]. EAT protects the coronary arteries mechanically and metabolically, as it buffers the torsion generated by the arterial pulse and cardiac contraction, and protects the myocardium from high concentrations of circulating inflammatory and pathogenic substances [20]. Also, EAT serves as a source of fatty acids for the myocardium during periods of high demand, as the heart is nurtured by fatty acids through *β*‐oxidation [21].

BAT is present in the cervical, supraclavicular, axillary, and paravertebral regions of adult mammals and is specialised in energy expenditure. BAT adipocytes are characterised by multiple small lipid droplets and many mitochondria, needed to release energy as heat in a non‐shivering thermogenesis process [22]. BAT exclusively expresses the mitochondrial protein Uncoupling Protein 1 (UCP‐1), which blocks ATP synthesis and dissipates energy as heat [23]. This thermogenic capacity makes BAT an interesting tissue to fight the complications of human obesity [24, 25].

Canonical WNT signalling is an evolutionarily conserved pathway needed in cellular proliferation and morphogenesis, the maintenance of tissue homeostasis, and the regulation of developmental processes [26]. The pathway is activated by the binding of canonical WNT ligands to membrane‐bound low‐density lipoprotein‐related proteins 5 and 6 (LRP5 / LRP6) and to Frizzled coreceptors, inducing the stabilisation and accumulation of *β*‐catenin in the cytoplasm that will translocate into the nucleus to activate the transcription factors TCF/Lef1, which regulate the expression of canonical WNT target genes [27].

Activation of canonical WNT signalling in preadipocytes by overexpression of the WNT ligands Wnt1 or Wnt10a, gain‐of‐function mutations in *β*‐catenin or pharmacological inhibition of the WNT inhibitor GSK3β (glycogen synthase kinase 3 beta) blocks adipogenesis by suppressing the expression of the adipogenic transcription factors PPARγ (peroxisome proliferator‐activated receptor gamma) and C/EBPα (CCAAT/enhancer‐binding protein alpha) [28]. Also, inhibition of WNT signalling in preadipocytes using secreted Frizzled‐Related protein 1 and 2 (sFRP1 and sFRP2) soluble inhibitors or enhanced expression of the negative regulator Axin results in spontaneous adipogenic differentiation, indicating that canonical WNT signalling and *β*‐catenin activity are important repressors for adipocyte differentiation [29, 30]. Furthermore, during mesenchymal stem cell differentiation, canonical WNT signalling stimulates the differentiation of multipotent cells into pre‐osteoblastic cells, blocking differentiation into pre‐adipocytic cells [31]. Taken together, these results suggest that in early stages of mesenchymal stem cell differentiation, the canonical WNT pathway is a repressor of adipose tissue generation. In contrast, the role of WNT signalling in terminally differentiated adipocytes is less understood, as the adipocyte‐specific abrogation of a transcription factor activated through canonical WNT signalling (Tcf7l2) leads to subcutaneous fat hypertrophy, impaired glucose tolerance, and insulin resistance [32, 33]. Due to the complexity of the canonical WNT signalling and the differences between the experimental models used to study obesity, the exact involvement of the canonical WNT pathway in the regulation of adipose tissue functions is still under investigation.

Mice and human patients with gain‐of‐function mutations in LRP5 exhibit a high‐bone mass (HBM) phenotype [34, 35] while loss‐of‐function mutations lead to osteoporosis [36], which in a small study with 12 subjects from two different families was also coupled with increased prevalence of Type‐2 diabetes [37]. Also, patients carrying LRP5 gain‐of‐function mutations show increased lower‐body fat, enhanced insulin sensitivity, and a lower inflammatory profile in subcutaneous adipocytes compared to non‐LRP5‐related HBM patients, indicating a role for canonical WNT signalling in modulating human fat distribution [38]. Furthermore, the expression of the rs559083 allele, associated with reduced LRP5 function, correlates with increased upper‐body fat accumulation [38]. In mice, loss of LRP5 expression in osteoblasts induces a loss of insulin sensitivity and ectopic lipid accumulation during high‐fat diet treatments [39]. *Lrp5* ^*−/−*^ mice pre‐adipocytes show impaired DNA repair, which results in increased inflammation in gluteal cells and a reduced ability of SCAT to proliferate [40].

Because weight gain induces adipose tissue growth, we hypothesised that mice fed high cholesterol diets have different adipose tissue distribution than mice fed a normocholesterolemic diet and that LRP5 could be involved in adipose tissue expansion. The roles of LRP5 in WAT and BAT mice adipose tissue depots were analysed and showed that LRP5‐dependent canonical WNT signalling is involved in insulin sensitivity, adipose tissue proliferation, and differentiation. Furthermore, LRP5 expression is needed for the infiltration of immune cells into the adipose tissue and for the triggering of inflammatory processes.

## 2 Results

### 2.1 Reduced Adipose Tissue Growth in *Lrp5* ^*−/−*^ Mice

**FIGURE 1:**
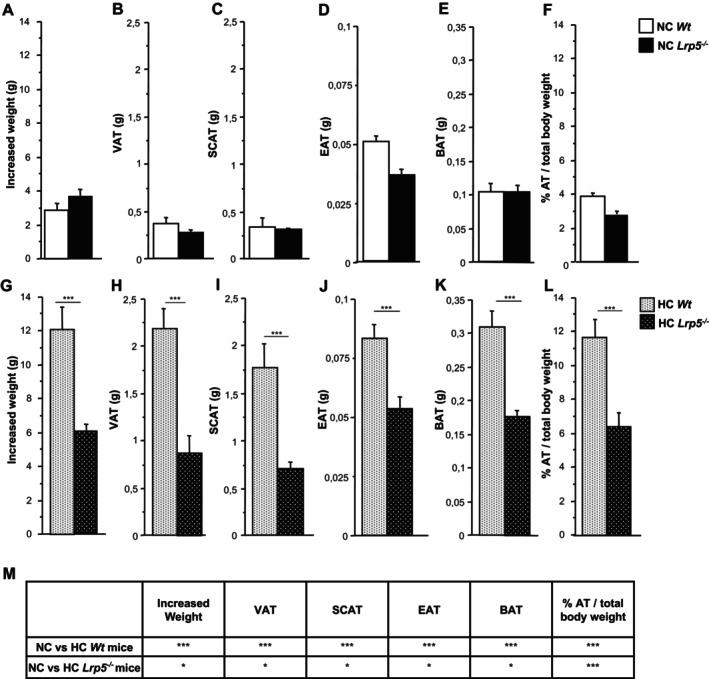
Mice body weight and mice adipose tissue weight. (A) *Wt* and *Lrp5* ^*−/−*^ mice weight gain after 8 weeks of NC diets. (B) VAT, (C) SCAT, (D) EAT, and (E) BAT weights from *Wt* and *Lrp5* ^*−/−*^ mice at sacrifice. (F) *Wt* and *Lrp5* ^*−/−*^ mice total adipose tissue weight against mice total body weight in NC mice. (G) *Wt* and *Lrp5* ^*−/−*^ mice weight gain after 8 weeks of HC diet feeding. (H) VAT, (I) SCAT, (J) EAT, and (K) BAT weights from *Wt* and *Lrp5* ^*−/−*^ mice at sacrifice. (L) *Wt* and *Lrp5* ^*−/−*^ mice total adipose tissue weight against mice total body weight in HC mice. (M) Significance between NC vs. HC *Wt* mice and NC vs. HC *Lrp5* ^*−/−*^ mice. *n* = 8–12 mice/group. *p < 0.05, ***p < 0.001.

*Wt* and *Lrp5* ^*−/−*^ mice were fed a normocholesterolemic (NC) or a hypercholesterolemic (HC) diet for 8 weeks, when mice were sacrificed and adipose tissue samples, including VAT, SCAT, EAT and BAT, were obtained. *Wt* and *Lrp5* ^*−/−*^ mice fed NC diets showed similar weight increase at sacrifice (Figure 1A). Adipose tissue weights for VAT (Figure 1B), SCAT (Figure 1C), EAT (Figure 1D) and BAT (Figure 1E) and the ratio between adipose tissue weight and total body weight was also similar in both genotypes (Figure 1F). The role of HC on mice's total body weight and adipose tissue weight was then analysed. After hypercholesterolemic feeding, HC *Wt* mice show a 9 g gain in total body weight compared to their NC littermates, while HC *Lrp5* ^*−/−*^ mice weight increased only by 2.5 g compared to NC *Lrp5* ^*−/−*^ mice (Figure 1A,G,M). Adipose tissue weights were increased after HC feeding in *Wt* and in *Lrp5* ^*−/−*^ mice (Figure 1H,K,M). The ratio between adipose tissue weight and total body weight showed increased fat accumulation in HC *Wt* mice compared to HC *Lrp5* ^*−/−*^ mice, indicating that mice without LRP5 accumulate less fat after high‐fat diets (Figure 1L).

Serum cholesterol levels in HC *Lrp5* ^*−/−*^ mice are significantly elevated compared to HC *Wt* mice ([41, 42, 43, 44]; Figure S1A). NC *Lrp5* ^*−/−*^ mice show lower baseline cholesterol levels than NC *Wt* mice, and the diet‐induced increase in serum cholesterol is significantly greater in *Lrp5* ^*−/−*^ mice compared to *Wt* mice (Figure S1A). Therefore, HC *Lrp5* ^*−/−*^ mice show increased serum cholesterol levels and impaired adipose tissue growth. Further analysis revealed that the exacerbated hypercholesterolemia in HC *Lrp5* ^*−/−*^ mice is primarily driven by elevated LDL‐cholesterol levels (Figure S1B) with no significant changes in HDL‐cholesterol (Figure S1C).

### 2.2 Increased LRP5 and LRP1 Expression in Hypercholesterolemic Mice

**FIGURE 2:**
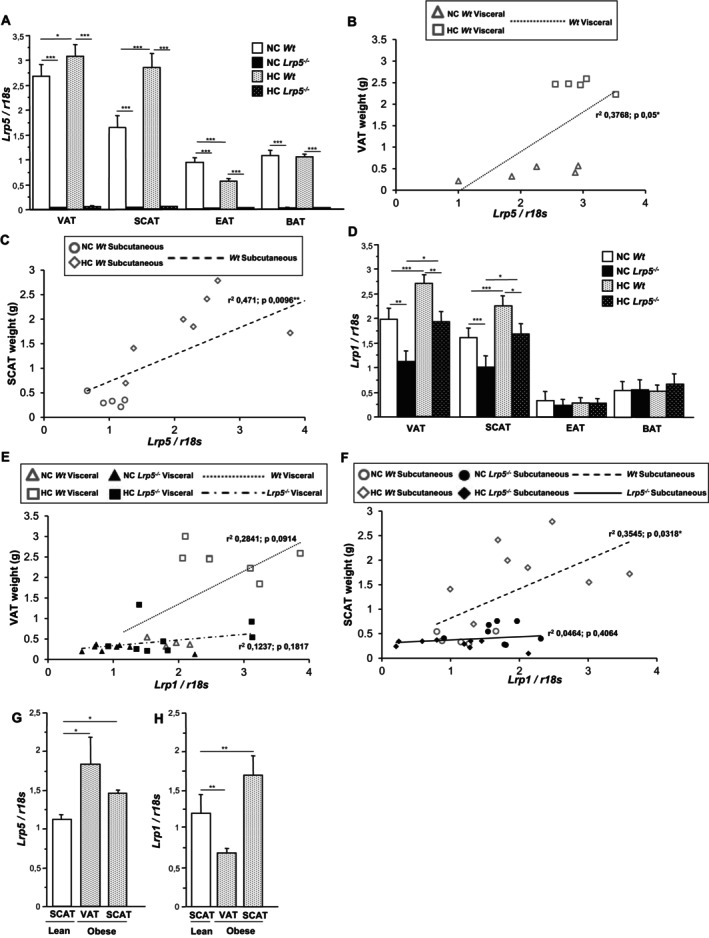
Low‐density lipoprotein receptors gene expression in mice and humans. (A) *Lrp5* gene expression in adipose tissue of *Wt* and *Lrp5* ^*−/−*^ mice fed NC or HC diets. Regression analysis for (B) *Lrp5* levels in VAT vs. VAT weight in *Wt* mice and (C) *Lrp5* expression in SCAT vs. SCAT weights in *Wt* mice. (D) *Lrp1* gene expression in adipose tissue of NC/HC *Wt* and *Lrp5* ^*−/−*^ mice. Regression analysis for (E) *Lrp1* levels in VAT vs. VAT weights in *Wt* and *Lrp5* ^*−/−*^ mice and (F) *Lrp1* levels in SCAT vs. SCAT weights in *Wt* and *Lrp5* ^*−/−*^ mice. *n* = 8–12 mice/group. (G) *Lrp5* and (H) *Lrp1* gene expression in adipose tissue of lean and obese patients. *n* = 3–5 subjects/group. *p < 0.05, **p < 0.01, ***p < 0.001.

Given that LRP1 and LRP5 are lipoprotein receptors known to mediate cellular lipid uptake from the circulation [44, 45, 46, 47], we sought to investigate their gene expression regulation in adipose tissue in response to high‐fat diet feeding. VAT and SCAT from *Wt* mice fed high cholesterol diets (HC) show increased *Lrp5* expression levels compared to *Wt* mice fed a normocholesterolemic (NC) diet (Figure 2A). Interestingly, *Lrp5* expression levels are downregulated in EAT after HC diets while they remain constant in BAT. *Lrp5* expression is undetectable in adipose tissues from *Lrp5* ^*−/−*^ mice (Figure 2A). Regression analyses between VAT (Figure 2B) and SCAT (Figure 2C) weight and *Lrp5* gene expression in *Wt* mice show a positive correlation, indicating that there is more *Lrp5* expression in VAT and SCAT from HC mice than in NC *Wt* mice. Expression of *Lrp1*, known to contribute to lipoprotein internalisation, is increased in VAT and SCAT from HC *Wt* and HC *Lrp5* ^*−/−*^ mice compared to NC *Wt* and NC *Lrp5* ^*−/−*^ mice, respectively, revealing that diets induce similar *Lrp1* upregulation in mice of both genotypes (Figure 2D). *Lrp5* expression levels in EAT and BAT are lower than those of VAT and SCAT and are not modified by diets or genotype (Figure 2D). *Lrp1* levels in VAT and SCAT of *Lrp5* ^*−/−*^ mice are lower than in *Wt* mice, independent of diets, indicating that *Lrp1* expression is downregulated in the absence of LRP5 expression in these tissues. A significant correlation between tissue (VAT and SCAT) weight and *Lrp5* expression is observed in *Wt* mice but not in *Lrp5* ^*−/−*^ mice, suggesting a role for LRP1 in adipocyte growth in HC *Wt* mice (Figure 2E,F).

### 2.3 Increased LRP5 and LRP1 Expression in Obese Patients

To determine the translational relevance of our murine results, lipoprotein receptors' expression was analysed in adipose tissues of obese and lean human individuals. VAT and SCAT from obese patients show increased *Lrp5* gene expression levels compared to SCAT from lean patients (Figure 2G). *Lrp1* expression is increased in SCAT from obese patients compared to SCAT obtained from lean patients (Figure 2H). Interestingly, *Lrp1* expression levels in VAT from obese patients are low, suggesting a minor role for LRP1 in lipid metabolism in VAT from obese patients (Figure 2H).

### 2.4 Adipocyte Markers Are Increased in Hypercholesterolemic Mice

**FIGURE 3:**
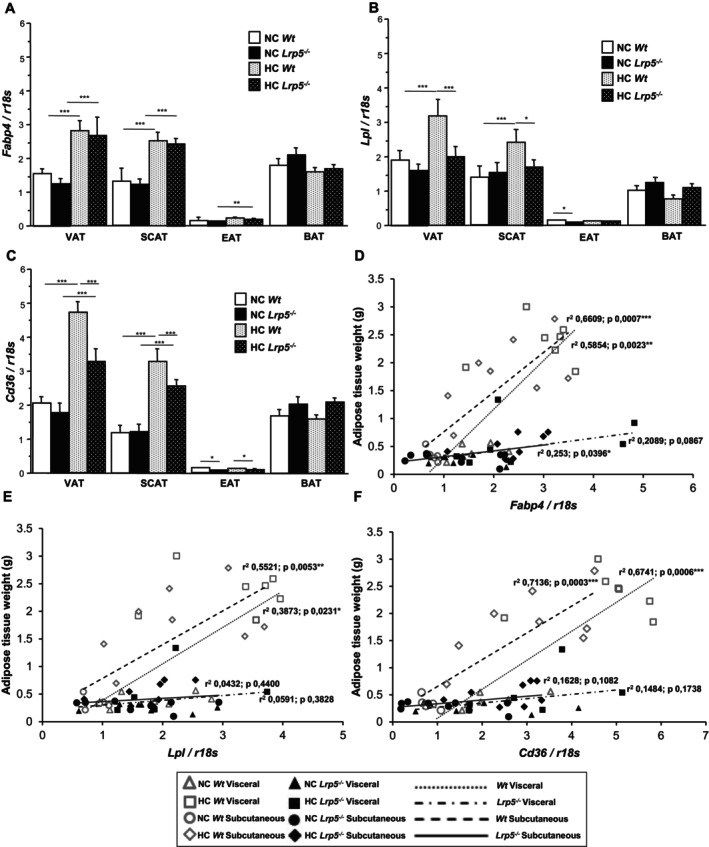
Adipocyte markers expression in mice. (A) *Fabp4*, (B) *Lpl*, and (C) *Cd36* expression levels in adipose tissues of NC/HC *Wt* and *Lrp5* ^*−/−*^ mice. Regression analysis of (D) *Fabp4*, (E) *Lpl*, and (F) *Cd36* gene expression in VAT and SCAT vs. VAT and SCAT weights from *Wt* and *Lrp5* ^*−/−*^ mice. *n* = 8–12 mice/group. *p < 0.05, **p < 0.01, ***p < 0.001.

A key feature of adipose tissue expansion is the upregulation of adipocyte maturation markers and lipid processing proteins. To assess adipogenic capacity, we evaluated the ability of *Wt* and *Lrp5* ^*−/−*^ mice to generate new mature adipose tissue. VAT and SCAT from HC *Wt* and HC *Lrp5* ^*−/−*^ mice show increased expression of the adipocyte marker *Fabp4*, suggesting increased presence of mature adipocytes (Figure 3A). *Fabp4* expression is very low in EAT and remains constant in BAT independently of dietary treatments or genotype (Figure 3A). *Lpl* gene expression levels are increased in VAT and SCAT from HC *Wt* mice compared to NC *Wt* mice, while they remain invariable in *Lrp5* ^*−/−*^ mice (Figure 3B). *Cd36* expression levels are upregulated by HC diets in *Wt* and *Lrp5* ^*−/−*^ mice, suggesting increased fatty acid absorption in HC mice adipocytes (Figure 3C). Similar to *Fabp4*, *Lpl* and *Cd36* expression levels in EAT are very low compared to the other adipose tissues, and *Lpl and Cd36* expression levels in BAT are moderately high and are not modified by diets or genotype (Figure 3B,C). Regression analyses between VAT and SCAT weight and *Fabp4*, *Lpl* and *Cd36* expression show significant correlation in *Wt* mice but not in *Lrp5* ^*−/−*^ mice, suggesting impaired ability to trigger the expression of mature adipocyte markers in the LRP5‐deficient adipose tissue in response to hypercholesterolemia (Figure 3D–F).

### 2.5 Macrophage Markers Are Reduced in *Lrp5* ^*−/−*^ Mice Adipose Tissues

**FIGURE 4:**
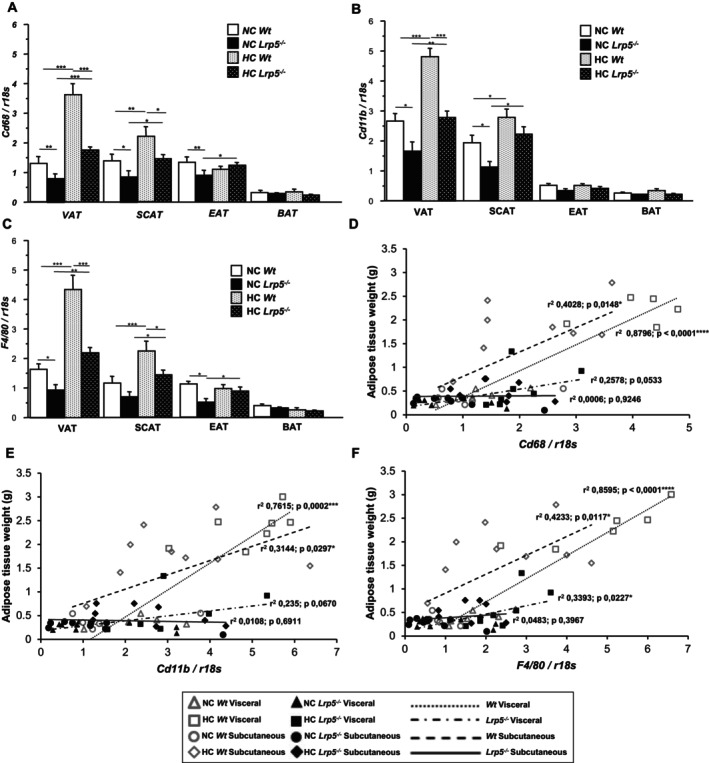
Macrophage markers in mice. (A) *Cd68*, (B) *Cd11b*, and (C) *F4/80* genetic levels in adipose tissues of NC/HC *Wt* and *Lrp5* ^*−/−*^ mice. Regression analysis of (D) *Cd68*, (E) *Cd11b*, and (F) *F4/80* levels in VAT and SCAT vs. VAT and SCAT weights from *Wt* and *Lrp5* ^*−*^ */* ^*−*^ mice. *n* = 8–12 mice/group. *p < 0.05, **p < 0.01, ***p < 0.001.

A critical aspect of adipose tissue expandability during hypercholesterolemia involves the associated inflammatory response, characterised by immune cell infiltration into the tissue microenvironment. To analyse the inflammatory profile of the different adipose tissues, we characterised gene expression levels of well‐defined macrophage markers. Expression levels of *Cd68, Cd11b*, and *F4/80* were upregulated in VAT and SCAT of HC animals compared to NC mice independently of genotype (Figure 4A–C). However, this upregulation only showed a significant correlation between gene expression and VAT and SCAT weights in *Wt* mice, indicating reduced presence of macrophages in *Lrp5* ^*−/−*^ mice adipose tissues (Figure 4D–F). Indeed, NC *Lrp5* ^*−/−*^ mice show less *Cd68, Cd11b*, and *F4/80* expression levels in white adipose tissues compared to NC *Wt* mice, indicating that *Lrp5* ^*−/−*^ mice have fewer resident macrophages. Gene expression of macrophage markers in BAT is extremely low, revealing a very small resident macrophage population (Figure 4A–C).

### 2.6 Pro‐Inflammatory Profile in Mice Adipose Tissues

**FIGURE 5:**
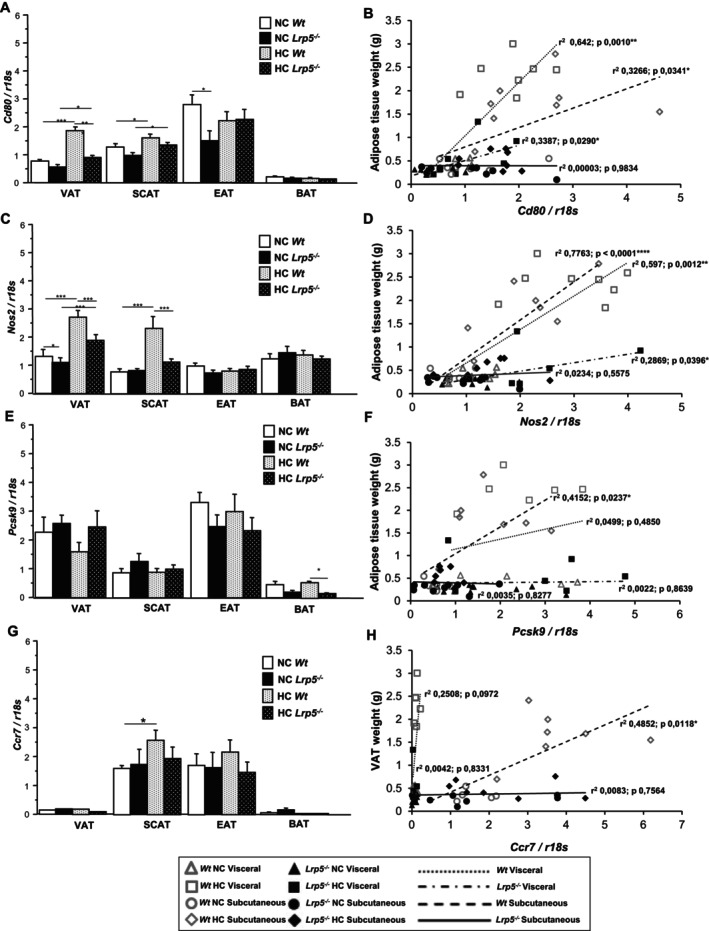
Pro‐inflammatory markers in mice adipose tissues. (A) *Cd80*, (C) *Nos2*, (E) *Pcsk9*, and (G) *Ccr7* gene expression in adipose tissues of *Wt* and *Lrp5* ^*−/−*^ mice fed NC or HC diets. Regression analysis of (B) *Cd80*, (D) *Nos2*, (F) *Pcsk9*, and (H) *Ccr7* gene expression in VAT and SCAT vs. VAT and SCAT weights in *Wt* and *Lrp5* ^*−*^ */* ^*−*^ mice. *n* = 8–12 mice/group. *p < 0.05, **p < 0.01, ***p < 0.001.

To better define the role of the inflammatory cells in the different adipose tissues, we further studied their inflammatory profile. NC *Wt* and NC *Lrp5* ^*−/−*^ mice show similar expression levels of the pro‐inflammatory marker *Cd80* in VAT and SCAT (Figure 5A). EAT shows the highest *Cd80* expression, while *Cd80* expression in BAT is almost undetectable (Figure 5A). *Cd80* expression levels correlated with VAT and SCAT weight in *Wt* mice but not in *Lrp5* ^*−/−*^ mice, indicating that there are fewer pro‐inflammatory cells in *Lrp5* ^*−/−*^ mice adipose tissues (Figure 5B).

Adipose tissue's pro‐inflammatory profile was further assessed by the analysis of *Nos2* and *Pcsk9* levels. After HC diets, *Nos2* gene expression is increased in VAT of both HC *Wt* and HC *Lrp5* ^*−/−*^ mice, indicating increased inflammation in HC‐fed mice compared to NC mice (Figure 5C). *Nos2* expression in EAT and BAT is not modified by diets or genotype (Figure 5C). *Nos2* expression significantly correlated with mice VAT weight in both *Wt* and *Lrp5* ^*−/−*^ mice, but the slope was steeper in *Wt* mice, suggesting more inflammation (Figure 5D). Similar to *Cd80*, *Nos2* genetic levels correlated with SCAT weight in *Wt* mice but not in *Lrp5* ^*−/−*^ mice (Figure 5D). These results show a higher pro‐inflammatory profile in VAT and SCAT of *Wt* mice triggered by adipose tissue expansion following hypercholesterolemic feeding.

VAT and EAT have relatively high *Pcsk9* gene expression levels independent of genotype or dietary treatments, while SCAT and BAT have relatively low *Pcsk9* expression (Figure 5E). Interestingly, HC diets do not modify *Pcsk9* expression, suggesting that, in the adipose tissues, *Pcsk9* is not regulated by lipids. Additionally, *Pcsk9* gene expression remains constant in *Wt* and *Lrp5* ^*−/−*^ mice, suggesting that LRP5 is not involved in *Pcsk9*'s adipose tissue gene expression regulation. Regression analyses show that fat mass and *Pcsk9* expression only correlated in SCAT of *Wt* animals, but not in VAT (Figure 5F), indicating that *Pcsk9* levels increased after adipocyte proliferation induced by high‐fat diets only in SCAT from *Wt* mice.

*Ccr7* (a lymphocyte and dendritic cell marker) expression is almost undetectable in VAT and BAT of *Wt* and *Lrp5* ^*−/−*^ mice. SCAT and EAT express *Ccr7* independent of genotype (Figure 5G). Furthermore, regression analyses show that *Ccr7* expression only correlates with the SCAT mass of *Wt* mice, indicating high lymphocyte infiltration induced by adipose tissue increase in *Wt* but not in *Lrp5* ^*−/−*^ mice (Figure 5H).

### 2.7 Anti‐Inflammatory Profile Is Preserved in *Lrp5* ^*−/−*^ Mice Adipose Tissues

**FIGURE 6:**
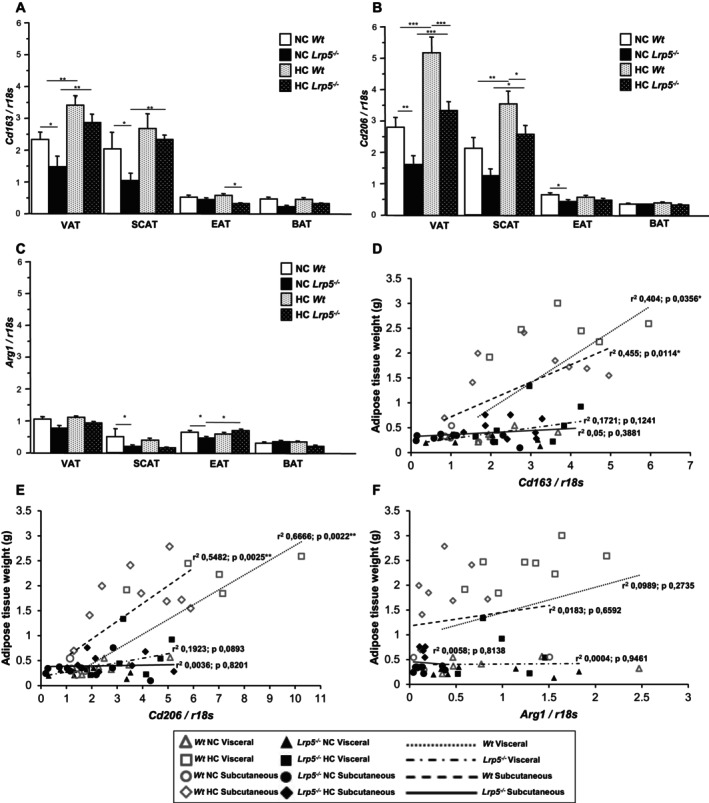
Anti‐inflammatory markers in mice adipose tissues. (A) *Cd163*, (B) *Cd206*, and (C) *Arg1* gene levels in adipose tissues of *Wt* and *Lrp5* ^*−/−*^ mice fed NC or HC diets. Regression analysis of (D) *Cd163*, (E) *Cd206*, and (F) *Arg1* levels in VAT and SCAT vs. VAT and SCAT weights in *Wt* and *Lrp5* ^*−/−*^ mice. *n* = 8–12 mice/group. *p < 0.05, **p < 0.01, ***p < 0.001.

To better understand the inflammatory process in adipose tissues, the anti‐inflammatory macrophage markers *Cd163, Cd206*, and *Arginase 1* (*Arg1*) were analysed. VAT and SCAT of *Wt* mice show higher *Cd163* expression than *Lrp5* ^*−/−*^ mice (Figure 6A). *Cd206* gene expression levels are also increased in VAT and SCAT of *Wt* mice compared to *Lrp5* ^*−/−*^ mice (Figure 6B). *Cd163* and *Cd206* expression levels in EAT and BAT are very low and do not increase after HC treatments (Figure 6A,B). Finally, *Arg1* gene expression analyses show a consistently low expression in all adipose tissues (Figure 6C). *Cd163* and *Cd206* expression levels correlated with both VAT and SCAT weights in *Wt* but not in *Lrp5* ^*−/−*^ mice, suggesting a reduction of anti‐inflammatory macrophage infiltration in VAT and SCAT after adipose tissue expansion (Figure 6D–F).

## 3 Discussion

The role of canonical WNT signalling in adipocytes is still under debate. LRP5 is involved in cell fate determination as canonical WNT signalling promotes osteoblastic rather than adipocytic differentiation in human mesenchymal stem cells [48]. Nonetheless, nuclear *β*–catenin–TCF/Lef1 complex can interact with the transcription factor p300, initiating adipocyte differentiation [49]. Also, the signals that lead to the promotion/inhibition of adipogenesis by the canonical WNT pathway are still not fully known. For example, Wnt5a, a canonical ligand inhibitor, is a negative regulator of adipogenesis, while vascular endothelial growth factor (VEGF), a target of the canonical WNT signalling, is closely involved in the growth and proliferation of adipocytes [50]. Our results support a role for LRP5 in the regulation of proliferation in mouse adipose tissues. Indeed, *Lrp5* ^*−/−*^ mice are leaner than *Wt* mice after HC diets. Furthermore, adipose tissue weight at sacrifice is also higher in HC *Wt* mice than in HC *Lrp5* ^*−/−*^ mice, indicating that mice without LRP5 accumulate less fat after high‐fat diets.

LRP1 mediates the internalisation of chylomicron remnants and LDL‐bound LPL in the liver [51]. When adipocytes are stimulated by insulin, LRP1‐mediated uptake of triglycerides and cholesteryl esters increases, together with LPL expression [52]. Interestingly, *Lrp1* and *Lpl* showed similar expression levels in *Wt* mice, and their expression increased after HC diets, when blood insulin concentration is higher. Loss of LRP5 expression in the adipose tissue significantly reduced *Lrp1* and *Lpl* expression, suggesting that tissue sensitivity to insulin might be reduced, resulting in reduced fat internalisation and lower weight gain in HC *Lrp5* ^*−/−*^ mice. *Cd36* gene expression levels are also low in HC *Lrp5* ^*−/−*^ mice compared to HC *Wt* mice. CD36 is a high‐affinity receptor for circulating long‐chain fatty acids that contribute to lipid accumulation in adipose tissues; therefore, it is an established marker for adipocytes [53, 54]. Lack of CD36 expression has been associated with insulin resistance [55, 56]. Human volunteers with low insulin sensitivity show decreased gene expression of LRP5, Wnt10b, Frizzled‐1, Frizzled‐8, and *β*‐catenin in white adipose tissue, indicating that an active canonical WNT pathway is needed for correct insulin sensitivity [57]. A crosstalk between WNT signalling and insulin signalling, where LRP5 is a coreceptor of both pathways, leading to the phosphorylation of Akt, ERK1/2, and GSK3β in undifferentiated adipocytes, has also been demonstrated [58]. Hence, previous reports support our hypothesis that *Lrp5* ^*−/−*^ mice adipocytes might have a defective response to insulin stimulation during hypercholesterolemia; however, additional experiments need to be performed to unveil the molecular mechanisms that control the canonical WNT‐insulin signalling crosstalk.

FABP4 belongs to the fatty acid binding protein family that regulates lipid trafficking [59, 60]. It accounts for 1% of all soluble proteins in WAT and indicates the presence of mature adipocytes [61]. High‐fat diets increase adipocyte *Fabp4* gene expression in WAT in mice and humans [62]. *Fabp4* expression was elevated in HC mice compared to NC mice, regardless of genotype. This suggests that the adipose tissue is able to generate fully differentiated adipocytes after HC diets in both *Wt* and *Lrp5* ^*−/−*^ mice. Hence, the observed differences in total body weight and adipose tissue weights between groups are unlikely to be solely explained by impaired adipocyte differentiation due to LRP5 deficiency. Nevertheless, regression analyses show that *Fabp4* expression does not correlate with adipose tissue weight in *Lrp5* ^*−/−*^ mice. This suggests a FABP4‐independent involvement in adipose tissue expandability. In vitro studies have demonstrated that intracellular FABP4 overexpression in the adipocyte progenitor cell line 3 T3‐L1 leads to reduced CD36 membrane expression, reduced lipid accumulation in fat cells, decreased adipocyte size, and the formation of new adipocytes [63]. However, in in vivo obesity models, FABP4 is vastly expressed extracellularly, leading to harmful events in the liver, the cardiovascular system, and the pancreas [64, 65]. Given that *Fabp4* expression does not correlate with adipose tissue weight in *Lrp5* ^*−/−*^ mice, it is likely that these mice exhibit a distinct regulatory mechanism in adipose tissue, potentially modifying their response to the systemic effects of FABP4 under hypercholesterolemic conditions. Further investigation is required to clarify the plausibility of this hypothesis.

Inflammatory cells play key roles in the differentiation process of the adipose tissue [17]. Increased monocyte/macrophage recruitment in HC *Wt* mice compared to NC *Wt* mice was observed by increased gene expression levels of *Cd68, Cd11b*, and *F4/80*. Interestingly, VAT and SCAT of HC *Lrp5* ^*−/−*^ mice show reduced expression of these markers compared to HC *Wt* mice, revealing a reduced population of inflammatory macrophages in HC *Lrp5* ^*−/−*^ mice. LRP5 is expressed in macrophages infiltrated into the vessel intima of human atherosclerotic plaques with a migratory function [66]. It is then plausible that *Lrp5* ^*−/−*^ mice macrophages show reduced migratory function and their infiltration into the adipose tissue is reduced; nevertheless, further experiments are required to properly identify adipose tissue infiltrating cells.

While in normal‐weight patients, adipose tissue macrophages (ATMs) show anti‐inflammatory properties, they shift to a pro‐inflammatory phenotype in obese patients [67]. Numerous studies have demonstrated a role for ATMs and inflammatory mediators in the impairment of insulin signalling pathways [68, 69, 70]. We observe a significant increase in the pro‐inflammatory macrophage marker *Cd80* in VAT of HC *Wt* compared to HC *Lrp5* ^*−/−*^ mice, indicating higher infiltration of inflammatory macrophages. Soluble PCSK9 participates in LDL‐ and VLDL‐derived triglyceride accumulation in mouse adipose tissues [71, 72]. Our results show that *Pcsk9* is mainly expressed in VAT and EAT. In humans, *Pcsk9* gene expression in VAT is associated with an elevated body mass index and a pro‐inflammatory profile, which is consistent with the elevated *Pcsk9* levels observed in mice [73]. Interestingly, the interaction observed between LRP5 and PCSK9 in lipid‐loaded human macrophages does not seem to occur in mouse adipose tissue [74]. Indeed, lipid‐loaded macrophages show low PCSK9 intracellular levels because it is transported to the plasma membrane and released to the extracellular milieu in a process that involves LRP5 [74]. However, because *Pcsk9* levels do not decrease after HC diets in mice adipose tissue, we hypothesise that this mechanism does not occur in adipocytes.

The anti‐inflammatory macrophage markers *Cd163* and *Cd206* increase their gene expression in VAT and SCAT of HC *Wt* and HC *Lrp5* ^*−/−*^ mice compared to their NC littermates. Interestingly, *Lrp5* ^*−/−*^ mice consistently show lower expression of macrophage anti‐inflammatory markers than *Wt* mice, suggesting an anti‐inflammatory role for LRP5 in VAT and SCAT and, in line with previous results, where LRP5 expression is associated with the anti‐inflammatory macrophage phenotype [75].

While low‐bone‐mineral‐density LRP5 alleles correlate with increased abdominal adiposity, gain‐of‐function LRP5 mutations are associated with a healthier lower‐body fat accumulation, suggesting a role for LRP5 and the canonical WNT pathway in the distribution of body fat [38]. LRP5 silencing in human VAT or SCAT adipocytes reduced their proliferation; however, only LRP5‐deficient SCAT adipocytes showed reduced differentiation [38]. Mutations in other canonical WNT signalling regulators, including the leucine‐rich repeat‐containing LGR4 (G protein‐coupled receptor‐4), ZNRF3 (the zinc and ring finger 3) or Rspo3 (R‐spondin 3) have been associated with adiposity as well. ZNRF3 is a transmembrane protein that regulates Frizzled receptor levels by ubiquitination, promoting its degradation and reducing canonical WNT signalling [76]. LGR proteins, on the other hand, promote canonical WNT signalling by stabilising the signal‐transducing LRP5/6‐Frizzled complex on the surface [77]. R‐spondin proteins are soluble ligands that bind to both receptors, inhibiting ZNRF3 and activating LGR [78]. A gain‐of‐function mutation in LGR4 is associated with increased central obesity, as human carriers are characterised by abdominal visceral fat accumulation [79]. In a meta‐analysis of GWAS for waist‐to‐hip ratio, the Rspo3 and the ZNRF3 loci were disclosed as regulators of waist‐to‐hip ratio; a rare SNP in Rspo3 correlated with an increased waist‐to‐hip ratio, while a SNP in ZNRF3 correlated with reduced waist‐to‐hip ratio [80]. In mice, *β*‐catenin was shown to have distinct activity in mature adipocytes and in adipocyte progenitors. *β*‐catenin expression in mature adipocytes contributed to adipocyte lipid metabolism preservation, but adipocyte progenitor *β*‐catenin expression induced a loss in adiposity as cells were differentiated into a fibroblastic‐like subtype [81]. These results indicate that canonical WNT signalling has distinct roles over the adipocyte differentiation process, inhibiting adipocyte differentiation in mesenchymal stem cells but preserving adipocyte lipid metabolism in mature adipose tissues. Our results postulate that not only did weight gain due to HC diets reduce in *Lrp5* ^*−/−*^ mice, but the amount of adipose tissue compared to total body weight was also significantly reduced, indicating a role for adipose tissue LRP5 in the regulation of obesity. In line with our results, loss of *Wntless* expression, a downstream effector of the canonical WNT pathway, protects mice from diet‐induced obesity and metabolic dysfunction [82]. It has also been reported that *β‐catenin* ^−/−^ mice are resistant to obesity induced by diet compared to control mice, while improving insulin sensitivity at the same time [83]. A recent study also demonstrated that the exclusive lack of *β*‐catenin expression in mice adipocytes fed a high‐fat diet exhibits decreased adiposity in mature adipose tissue, indicating that the pathway is a key regulator of mature adipocyte *de novo* lipogenesis and fatty acid desaturation [84]. Also, a potential role for stromovascular cells (mesenchymal stem cell progenitors located in the adipose tissue) to replace the loss of function in *β*‐catenin‐deficient differentiated adipocytes has been suggested, indicating a homeostatic role for canonical WNT signalling in WAT lipid metabolism [8]. Interestingly, results observed in *β‐catenin* ^−/−^ mice challenged with high‐fat diets support the results observed in *Lrp5* ^*−/−*^ mice, as HC *β‐catenin* ^−/−^ mice are also leaner than their *Wt* littermates, indicating reduced obesity when the canonical WNT signalling pathway is downregulated.

Of note, neither a high‐cholesterol diet nor LRP5 deficiency altered gene expression patterns in EAT or BAT. EAT plays a unique physiological role in cardiac protection and myocardial nourishment. Obesity‐related cardiovascular risk factors, including hypertension [85] and diet‐induced cardiac hypertrophy [86], highlight the importance of EAT adaptation. Our findings suggest that while EAT proliferation and volumetric expansion are necessary to maintain cardiac function during metabolic stress, its gene expression profile remains stable regardless of dietary challenge or LRP5 expression.

We observed HC diet‐induced downregulation of LRP5 expression in *Wt* mice EAT, contrasting with VAT and SCAT depots. Unlike these depots, EAT thickness shows no correlation with body mass index and only becomes an ectopic fat reservoir in late‐stage obesity [87]. In our study, the hypercholesterolemic mouse model exhibited moderate hypercholesterolemia (25% increase in serum LDL‐cholesterol) after the 8‐week experimental period. This metabolic challenge may have been insufficient to induce significant EAT expansion. The observed BAT mass differences between HC *Wt* and HC *Lrp5* ^*−/−*^ mice likely reflect systemic metabolic changes associated with total body weight gain.

Our study has several limitations. The study provides transcriptomic data without protein expression analysis. However, we believe that these RNA‐based findings are enough to sustain our conclusions. Additionally, we did not monitor caloric intake in individual mice during the feeding period. This measurement would have provided valuable insight into the relationship between energy consumption and the observed weight gain differences among experimental groups. Also, we provide data from an *Lrp5* ^*−/−*^ mouse model only. Similar analyses in other research models with a lack of expression of canonical WNT signalling members would be of great interest.

In conclusion, our results show that mice fed a high‐fat diet have different adipose tissue distribution than NC mice. Furthermore, we postulate a mechanism where LRP5 may play a role in adipose tissue growth by promoting tissue proliferation and insulin sensitivity while also enhancing macrophage's infiltrating capacity into the adipose tissue, which may contribute to the inflammatory process linked to growing adipose tissue.

## 4 Methods

### 4.1 Animal Protocols

Experimental procedures were reviewed and approved by the Institutional Animal Care and Use Committee of the Sant Pau Research Institute and authorised by the Animal Experimental Committee of the local government authority (Generalitat de Catalunya, authorization No. ICCC051/5422) in accordance to the Spanish Law (RD 53/2013) and the European Directive 2010/63/EU. Procedures were performed at the Animal Experimentation Service, ISO 9001:2015 certified. In addition, the investigation conforms to the Guide for the Care and Use of Laboratory Animals published by the US National Institutes of Health (NIH Publication No. 85–23, revised 1985), follows the ARRIVE guidelines (Animal Research: Reporting of In Vivo Experiments), and is committed to the 3Rs of laboratory animal research and consequently used the minimal number of animals to reach statistical significance.

*Lrp5* alleles were amplified by PCR of DNA extracted from tail biopsies. Primers sequence for PCR amplification were as follows: S17 (GGC TCG GAG GAC AGA CCT GAG), S23 (CTG TCA GTG CCT GTA TCT GTC C) and IRES31 (AGG GGC GGA ATT CGA TAG CT). *Wt* and *Lrp5* ^*−/−*^ mice were fed a normal chow diet for the first 10 weeks (NC, Tekland Diet, Harland Labs Berkeley, CA, USA) when they were divided into two groups to be fed a NC or a HC diet (42% fat; TD.88137, Harland Labs) for the following 8 weeks (8–12 mice per group). Mice were weighed weekly from 10 to 18 weeks.

At 18 weeks old, mice were terminally anesthetised by intraperitoneal injection of 1 mg/kg medetomidine and 75 mg/kg ketamine. Cardiac puncture was performed to obtain the maximum blood volume. Immediately after, fat depots were collected and frozen by immersion in liquid nitrogen for further processing. SCAT contained the fat depots in the inguinal and axillar areas; VAT contained the fat depots in the omental and mesenchymal areas; EAT contained the fat depots covering the upper region of the heart, and BAT contained the fat depots in the interscapular dorsal region of the mice. Before freezing, each adipose tissue mass was weighed.

Mice blood samples were collected at sacrifice by cardiac puncture, and the serum was isolated after centrifugation at 3000 rpm for 20 min at 4°C. Total cholesterol and HDL levels were quantified by enzymatic measurement using available commercial kits from GERNON Reagents and a spectrophotometer MC‐15 SOFT (RAL). Non‐HDL cholesterol was calculated with the formula “Total Cholesterol – HDL Cholesterol = Non‐HDL Cholesterol”.

### 4.2 Human Adipose Tissue Collection

SCAT and VAT were obtained via surgical resection from young individuals with morbid obesity (BMI > 40 kg/m^2^) who underwent bypass gastric surgery. Additionally, SCAT was collected from young individuals with normal weight (BMI < 25 kg/m^2^) who underwent abdominal lipectomy. The sample size for each group was 3–5 individuals. Informed consent was obtained from all donors, and the study protocol was approved by the Centro Medico Teknon Ethical Committee, which is in accordance with the principles of the Declaration of Helsinki.

### 4.3 Tissue Processing

Frozen tissue samples were disaggregated in liquid nitrogen, and 1 g of each sample was processed using an RNeasy Extraction Kit (Qiagen) to obtain pure RNA. RNA concentration and purity were determined using a NanoDrop ND‐1000 spectrophotometer (Nanodrop Technologies), and only samples with ratios 260/280 and 260/230 between 1.8–2.2 were considered acceptable. cDNA was synthesised using a cDNA Reverse Transcription kit (Qiagen). The resulting cDNA samples were amplified in a RT‐qPCR thermal cycler (Applied Biosystems) using the following probes from ThermoFisher: *Lrp5* (Mm00493187_m1 / Hs00182031_m1), *Lrp1* (Mm00464608_m1 / Hs00233856_m1), *Fabp4* (Mm00445878_m1), *Lpl* (Mm00434764_m1), CD36 (Mm00432403_m1), *Cd68* (Mm03047343_m1), *Cd11b* (Mm00434455_m1), *F4/80* (Mm00802529_m1), *Nos2* (Mm00440502_m1), *Cd80* (Mm00711660_m1), *Ccr7* (Mm00432608_m1), *Pcsk9* (Mm01263610_m1), *Arg1* (Mm00475988_m1), *Cd163* (Mm00474091_m1) and *Cd206* (Mm01329359_m1).

### 4.4 Statistical Analysis

Results are expressed as mean ± standard error of the mean. StatView statistical software was used for all the analyses. Comparisons among groups were performed using One‐way ANOVA analysis. Differences between groups were performed using Fisher's test. Statistical significance was considered when *p* < 0.05. Simple Regression analyses were performed with VAT or SCAT weight as the independent variable and gene expression corrected by r18s expression as the dependent variable. Correlation between variables was considered significant when *p* < 0.05.

## Author Contributions

**Aureli Luquero:** data curation (lead), formal analysis (lead), investigation (equal), methodology (lead), software (lead), validation (equal), visualization (supporting), writing – original draft (lead). **Noelia Pimentel:** investigation (supporting), methodology (supporting), software (equal), writing – review and editing (supporting). **Lina Badimon:** funding acquisition (equal), methodology (supporting), project administration (supporting), resources (equal), supervision (equal), validation (supporting), writing – review and editing (equal). **Gemma Vilahur:** conceptualization (supporting), funding acquisition (supporting), project administration (equal), validation (equal), writing – review and editing (supporting). **Maria Borrell‐Pages:** conceptualization (equal), data curation (supporting), formal analysis (equal), funding acquisition (lead), investigation (equal), methodology (supporting), project administration (lead), resources (lead), software (supporting), supervision (lead), validation (equal), visualization (lead), writing – original draft (supporting), writing – review and editing (lead).

## Ethics Statement

The procedures carried out during this study have been subjected to an evaluation process by ethical committees, both in the case of studies involving mice and in the collection of human samples. In the case of human samples, informed consent has also been obtained from each of the patients.

## Conflicts of Interest

The authors declare no conflicts of interest.

## Supporting information


**Figure S1.** Mice lipid profile. (A) Serum cholesterol levels in *Wt* and *Lrp5* ^*−/−*^ mice fed a NC or a HC diet. (B) Non‐HDL‐cholesterol and (C) HDL‐cholesterol in *Wt* and *Lrp5* ^*−/−*^ mice fed a NC or a HC diet. *n* = 28–36 mice/group. *p < 0.05; ***p < 0.005.

## Data Availability

All the information of the research is contained within the manuscript. Detailed data is available from the corresponding author upon reasonable demand.
