# Supplementary material for: Reduced Growth and Inflammation in Lrp5 −/− Mice Adipose Tissue
Source: J Cell Mol Med. 2025 Oct 13;29(19):e70670. doi: 10.1111/jcmm.70670 (PMC12516158; doi:10.1111/jcmm.70670)
Supplement: Supplementary file 1 — Figure S1. Mice lipid profile. (A) Serum cholesterol levels in Wt and Lrp5 −/− mice fed a NC or a HC diet. (B) Non‐HDL‐cholesterol and (C) HDL‐cholesterol in Wt and Lrp5 −/− mice fed a NC or a HC diet. n = 28–36 mice/group. *p < 0.05; ***p < 0.005. [file JCMM-29-e70670-s001.pptx]

## Slide 1
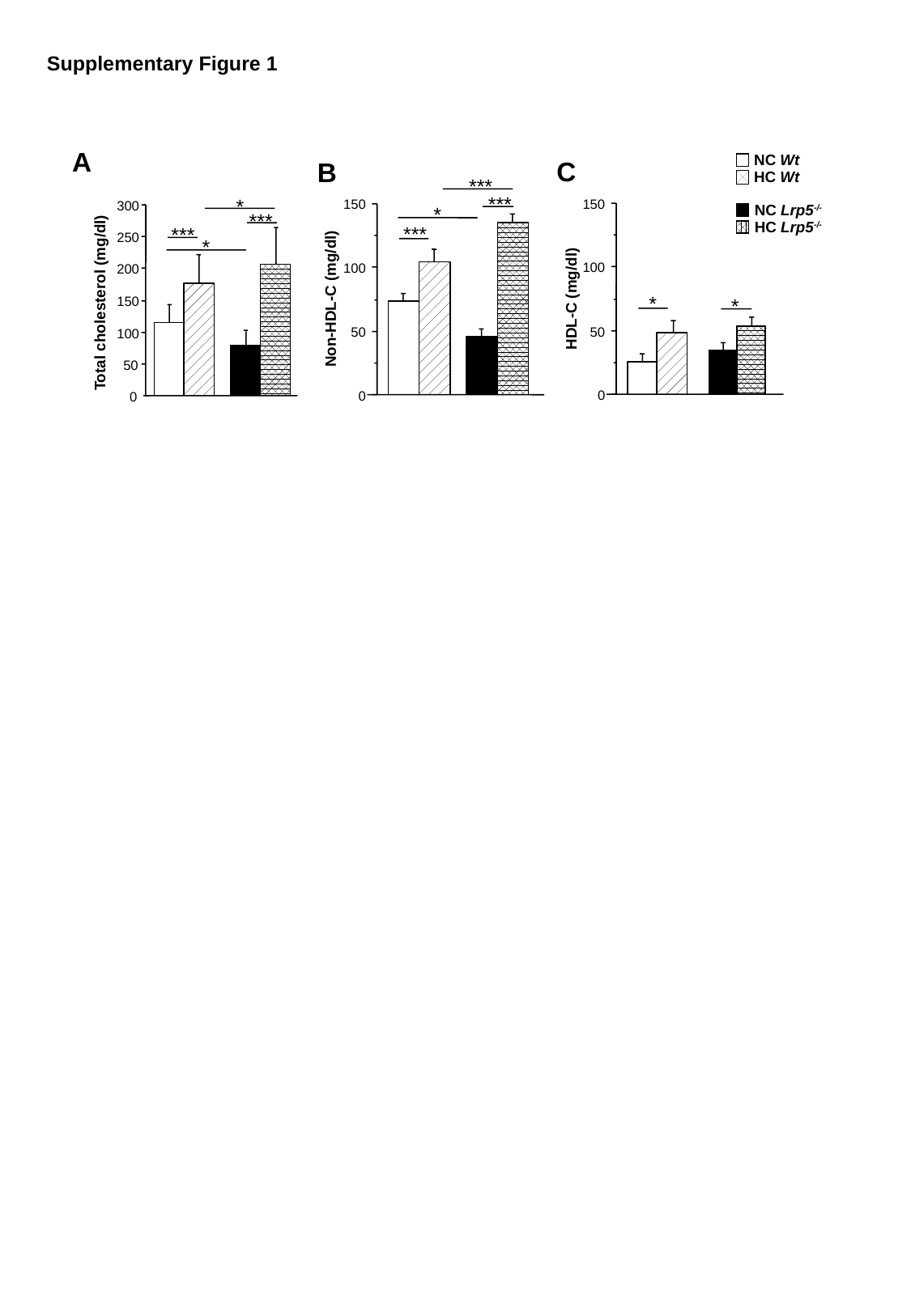

Supplementary Figure 1
A
NC Wt
HC Wt
NC Lrp5-/-
HC Lrp5-/-
C
B
***
***
*
150
150
*
300
***
***
***
*
250
100
100
200
*
*
HDL-C (mg/dl)
Non-HDL-C (mg/dl)
150
Total cholesterol (mg/dl)
50
50
100
50
0
0
0
